# Notes and Comments—A Curious Hypnotic Test

**Published:** 1890-05

**Authors:** 


### Notes and Comments—A Curious Hypnotic Test.

Dr. J. M. Charcot writes in the January *Forum:*

"The end I have ever held before my eyes, then, and which I hope I have never lost from view, is this: To study the hypnotic phenomena according to a strictly scientific method, and for this purpose to employ processes purely physical, and which can always be compared with one another, so that the results obtained by me may be vigorously tested by all observers who shall use the same processes under the same conditions.

"Take one example from among a thousand. I present to a woman patient in the hypnotic state a blank leaf of paper and say to her: 'Here is my portrait; what do you think of it? Is it a good likeness?' After a few moments hesitation, she answers: 'Yes, indeed, your photograph; will you give it to me?' To impress deeply in the mind of the subject this imaginary portrait, I point with my finger toward one of the four sides of the square leaf of paper, and tell her that my profile looks in that direction; I describe my clothing. The image being now fixed in her mind, I take that leaf of paper and mix it with a score of other leaves precisely like it. I then hand the whole pack to the patient, bidding her to go over them and let me know whether she finds among these anything she has seen before. She begins to look at the leaves one after the other, and as soon as her eyes fall upon the one first shown to her (I had made upon it a mark that she could not discern), forthwith she exclaimed: 'Look, your portrait!' What is more curious still, if I turn the leaf upside down, as soon as her eyes rest upon it she turns it over, saying that my photograph is on the obverse. I then convey to her the order that she shall continue to see my portrait on the blank paper even after the hypnosis has passed. Then I awaken her and again hand to her the pack of papers, requesting her to look over them. She handles them just as before, when she was hypnotized, and utters the same exaclamation: 'Look, your portrait!' If now I tell her she may retire, she returns to her dormitory, and her first care will be to show to her companions the photograph I have given her. Of course, her companions, not having received the suggestion, will see only a blank leaf of paper without any trace whatever of a portrait, and will laugh at our subject and treat her as visionary. Furthermore, this suggestion, this hallucination, will, if I wish, continue several days; all I have to do is to express the wish to the patient before awakening her.

"The foregoing experiments have been made hundreds of times by me and by others, and the fact can easily be substantiated; their objectivity is as complete as could be wished in researches of this kind. Hypnotism is directly amenable to our means of investigation, and must needs be an integral part of the known domain of science. To that goal our efforts ought to be directed."—*Boston Med. and Surg. Journal*, Jan. 23, 1890.

